# Symmetric spike timing-dependent plasticity at CA3–CA3 synapses optimizes storage and recall in autoassociative networks

**DOI:** 10.1038/ncomms11552

**Published:** 2016-05-13

**Authors:** Rajiv K. Mishra, Sooyun Kim, Segundo J. Guzman, Peter Jonas

**Affiliations:** 1 Institute of Science and Technology Austria, Am Campus 1, Klosterneuburg, A-3400, Austria

## Abstract

CA3–CA3 recurrent excitatory synapses are thought to play a key role in memory storage and pattern completion. Whether the plasticity properties of these synapses are consistent with their proposed network functions remains unclear. Here, we examine the properties of spike timing-dependent plasticity (STDP) at CA3–CA3 synapses. Low-frequency pairing of excitatory postsynaptic potentials (EPSPs) and action potentials (APs) induces long-term potentiation (LTP), independent of temporal order. The STDP curve is symmetric and broad (half-width ∼150 ms). Consistent with these STDP induction properties, AP–EPSP sequences lead to supralinear summation of spine [Ca^2+^] transients. Furthermore, afterdepolarizations (ADPs) following APs efficiently propagate into dendrites of CA3 pyramidal neurons, and EPSPs summate with dendritic ADPs. In autoassociative network models, storage and recall are more robust with symmetric than with asymmetric STDP rules. Thus, a specialized STDP induction rule allows reliable storage and recall of information in the hippocampal CA3 network.

## Abstract (web summary)

STDP is dependent on the timing of pre- and post-synaptic activity. Here, the authors describe a symmetric STDP induction rule at CA3-CA3 synapses, which induces LTP over a broad range of paring intervals. Modelling suggests that this STDP rule may enhance storage capacity and pattern completion in the CA3 cell network.

## Graphical abstract

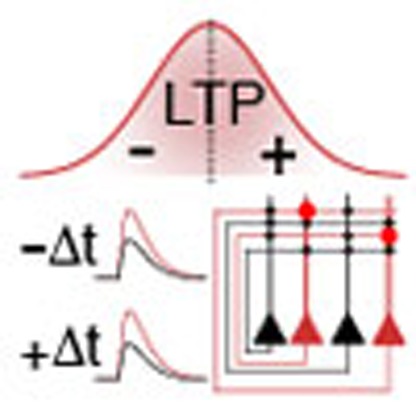

The hippocampal CA3 region plays a critical role in the storage, recall and processing of spatial and non-spatial information in the brain^1^. A hallmark property of the CA3 network is that recall can be triggered by partial or degraded cues, a phenomenon referred to as autoassociative recall or pattern completion^2^,^3^,^4^,^5^,^6^. Several lines of evidence suggest that CA3–CA3 recurrent synapses are the main subcellular correlates of pattern completion. First, the CA3–CA3 synaptic system is the most extensive synaptic system in the entire brain^7^, generating a connectivity matrix with potentially huge storage capacity. Second, CA3–CA3 synapses show robust long-term plasticity, rendering them suitable for sustained information storage^8^,^9^,^10^. Third, selective genetic deletion of *N*-methyl-D-aspartate (NMDA)-type glutamate receptors in CA3 pyramidal neurons impairs pattern completion, presumably by abolishing plasticity at CA3–CA3 synapses^11^. Finally, network models endowed with recurrent connectivity and Hebbian synaptic plasticity can reproduce storage, recall and pattern completion^2^,^3^,^4^,^5^,^6^,^12^,^13^.

Spike timing-dependent plasticity (STDP) is the primary candidate mechanism for the storage of information in autoassociative networks^4^,^6^,^14^. In conventional STDP, pre–postsynaptic pairing induces long-term potentiation (LTP), whereas pairing in reverse order triggers long-term depression (LTD)^15^,^16^,^17^,^18^,^19^,^20^. It is, however, unclear whether LTP with such induction rules would support storage and autoassociative recall of information in CA3 pyramidal neuron ensembles. Recent recordings from dendrites and axons of CA3 pyramidal neurons revealed that dendritic backpropagation of the action potential (AP) over a short distance is faster than axonal forward propagation over a longer distance^21^. Thus, synchronous activity of CA3 pyramidal neurons (for example, during network oscillations or mossy fibre activation) is expected to generate a post–presynaptic activity sequence, which would induce LTD, rather than LTP^15^,^16^,^17^. Furthermore, CA3–CA3 synapses are often located on CA3 pyramidal cell dendrites at a considerable distance from the soma (∼100–200 μm)^7^,^22^,^23^. STDP may be biased towards LTD under these conditions^20^,^24^,^25^,^26^. Thus, whether the plasticity properties of CA3–CA3 synapses are consistent with their proposed network functions remains unresolved.

Although associative plasticity has been demonstrated in CA3 pyramidal neurons in organotypic slice culture^9^,^10^, the plasticity properties in intact neuronal preparations have not been examined yet. We therefore experimentally determined the STDP induction rules at CA3–CA3 recurrent synapses in acute slices from 21- to 29-day-old rats. Surprisingly, pairing of excitatory postsynaptic potentials (EPSPs) and APs induced LTP, independent of temporal order. Consistent with these novel STDP induction properties, both pre–postsynaptic and post–presynaptic sequences led to efficient summation of spine [Ca^2+^] transients and dendritic depolarizations. When we incorporated different STDP induction rules into autoassociative network models, recall was substantially more robust in models with the novel induction rule. Thus, a specific LTP induction rule at CA3–CA3 synapses facilitates information storage and recall in the hippocampal CA3 network.

## Results

### A novel STDP induction rule at CA3–CA3 recurrent synapses

**Figure 1:**
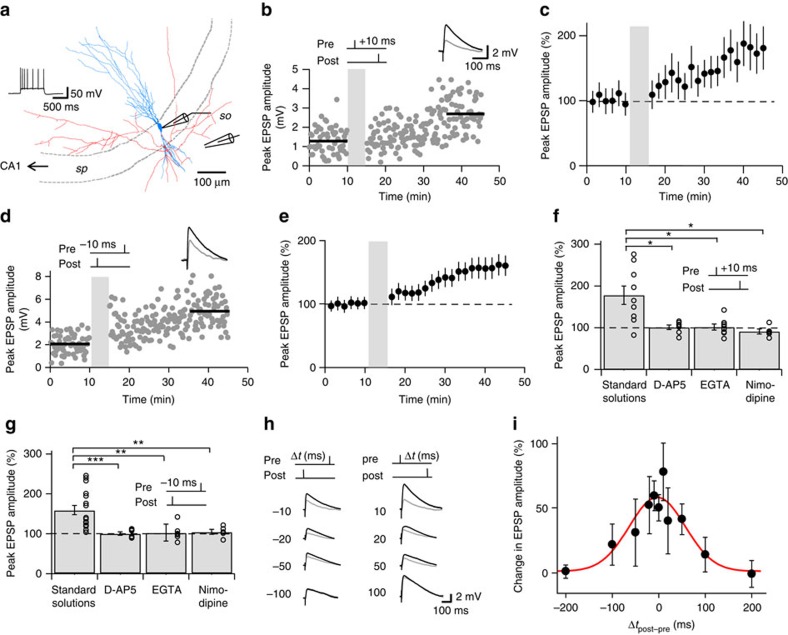
Noncanonical STDP induction rules at CA3–CA3 recurrent synapses. (**a**) Neurolucida reconstruction of a representative CA3 pyramidal neuron filled with biocytin. Inset shows AP phenotype (1-s current pulse, 630 pA); recording and stimulation pipettes are indicated schematically. Blue, soma and dendrites; red, axon; sp, *stratum pyramidale*; so, *stratum oriens*. (**b**,**c**) Pre–postsynaptic pairing induces LTP at CA3–CA3 recurrent synapses. Plot of compound EPSP peak amplitude against experimental time before and after pre–postsynaptic pairing (Δ*t*=+10 ms; vertical grey bars). Single-cell data (**b**) and mean data (**c**; 9 cells). (**d**,**e**) Post–presynaptic pairing also induces LTP. Similar plot as in **b**, **c**, but for post–presynaptic pairing (with Δ*t*=–10 ms). Single-cell data (**d**) and mean data (**e**; 17 cells). Insets in **b** and **d** show the average of 60 evoked EPSPs before (black) and 20–30 min after induction (grey). Scale bar in **b** also applies in **d**. In **c** and **e**, EPSP amplitude was normalized to the control value before LTP induction (dashed line). (**f**,**g**) Multiple Ca^2+^ sources were necessary for STDP induction. Summary bar graph shows the effects of 20 μM of the NMDA receptor antagonist D-AP5 (extracellular; 7 and 6 cells), 20 mM of the Ca^2+^ chelator EGTA (intracellular; 8 and 6 cells) and 10 μM of the L-type Ca^2+^ channel blocker nimodipine (extracellular; 5 and 5 cells, respectively) on pairing-induced LTP for pre–postsynaptic (**f**) and post–presynaptic sequences (**g**). Circles represent data from individual cells, bars indicate mean±s.e.m. LTP with both pairing paradigms was largely abolished by all manipulations. (**h**,**i**) A broad and symmetric STDP curve at CA3–CA3 synapses. Magnitude of potentiation induced by pre–postsynaptic and post–presynaptic pairing was plotted against pairing time interval Δ*t*. Representative average traces are shown in **h** (grey, before induction; black, 20–30 min after induction), plot of LTP magnitude (expressed as % increase over baseline) against Δ*t* is shown in **i** (69 cells total). Red curve, Gaussian function without offset fit to the data points. *, *P*<0.05; **, *P*<0.01; ***, *P*<0.001.

To examine whether the plasticity properties of CA3–CA3 recurrent synapses are consistent with their proposed function in storage and recall, we studied synaptic plasticity of these synapses in acute hippocampal slices from mature (21- to 29-day-old) rats (Fig. 1a; Supplementary Fig. 1; Methods and Table 1). Presynaptic axons were stimulated in the *stratum oriens*, whereas compound EPSPs were recorded in CA3 pyramidal cells in the whole-cell current-clamp recording configuration.

We first tested the effects of a pairing paradigm in which presynaptic stimulation was followed at a 10-ms interval by a postsynaptic current pulse (that is, a pre–postsynaptic sequence). This pairing protocol, repeated 300 times at a frequency of 1 Hz, induced a robust LTP. On average, the compound EPSP amplitude increased to 178.0±22.1% of control 20−30 min after LTP induction (9 cells, *P*=0.004; Fig. 1b,c and Table 1). We next compared the extent of pairing-induced LTP with LTP induced by high-frequency stimulation (HFS), a paradigm expected to maximally potentiate synapses^8^. Multiple rounds of HFS stimulation (four 100-Hz trains of 1-s duration, repeated every 10 s) induced a synaptic potentiation of 196.8±18.4% of control under identical conditions (14 cells, *P*=0.0002; Supplementary Fig. 2 and Table 1). Thus, the amount of pairing-induced LTP was comparable to that of HFS-induced LTP. LTP was associative, since neither EPSPs nor postsynaptic APs induced LTP when presented in isolation (isolated presynaptic stimulation: 102.8±2.9%, 6 cells, *P*=0.31; isolated postsynaptic stimulation: 95.7±4.4%; 6 cells, *P*=0.38; Supplementary Fig. 3). Thus, CA3–CA3 recurrent synapses exhibited an associative form of LTP, with an extent of potentiation comparable to that of the most efficient LTP induction paradigms.

Next, we tested pairing protocols with an inverse (that is, post–presynaptic) sequence (Fig. 1d,e). In contrast to our expectations, such a pairing protocol led to an increase of EPSP amplitude, to 159.3±11.5% of the control value (17 cells, *P*=0.00002; Fig. 1d,e). Next, we compared the mechanisms of STDP induction for LTP induced by pre–postsynaptic versus post–presynaptic sequences. Pairing-induced LTP was abolished by bath application of the NMDA receptor antagonist D-2-amino-5-phosphonovaleric acid (D-AP5). In the presence of 20 μM D-AP5, the extent of potentiation was 101.2±5.2% for the pre–postsynaptic sequence (7 cells, *P*=0.03; Fig. 1f) and 100.5±4.2 % for the post–presynaptic sequence (6 cells, *P*=0.001; Fig. 1g). Thus, NMDA receptor activation was necessary for LTP induction. Furthermore, pairing-induced LTP was abolished by 20 min prior intracellular loading with the Ca^2+^ chelator ethylene glycol-bis(2-aminoethylether)-*N*,*N*,*N*′,*N*′-tetraacetic acid (EGTA). In the presence of 20 mM EGTA in the pipette solution, the extent of potentiation was 101.8±7.2% for the pre–postsynaptic sequence (8 cells, *P*=0.02; Fig. 1f) and 103.0±9.0% with the post–presynaptic sequence (6 cells, *P*=0.01; Fig. 1g). Thus, a rise in postsynaptic Ca^2+^ concentration was necessary for LTP induction. Finally, pairing-induced LTP was inhibited by bath application of the L-type Ca^2+^ channel blocker nimodipine. In the presence of 10 μM nimodipine, the extent of potentiation was 91.1±6.0% for the pre–postsynaptic sequence (5 cells, *P*=0.04; Fig. 1f) and 105.0±6.1% for the post–presynaptic sequence (5 cells, *P*=0.01; Fig. 1g). Thus, activation of NMDA receptors and L-type Ca^2+^ channels was necessary for LTP induction by both pre–postsynaptic and post–presynaptic pairing (Table 1).

To further characterize the induction rules of STDP at CA3–CA3 cell synapses, we tested pairing protocols with different time intervals between pre- and postsynaptic stimulation (Fig. 1h,i). Plotting the extent of LTP against pairing time interval Δ*t* revealed that the STDP induction curve was nearly symmetric and surprisingly broad, with a maximum near Δ*t*=0 and a half-duration of 142 ms (Fig. 1i). Similar results were obtained at ∼22 and ∼33 °C. At near-physiological temperature (∼33 °C), the maximal amount of LTP (202.2±25.3% for Δ*t*=+10 ms; 6 cells) and the shape of the curve were comparable (half-duration 133 ms; Supplementary Fig. 4). In conclusion, CA3–CA3 recurrent synapses exhibited robust STDP with a temporally symmetric and broad induction window. Thus, the STDP induction rule at CA3–CA3 recurrent synapses was unique, differing from previously reported STDP rules for glutamatergic synapses in a variety of circuits^20^.

### Spine [Ca^2+^] summation may explain symmetric LTP induction

**Figure 2:**
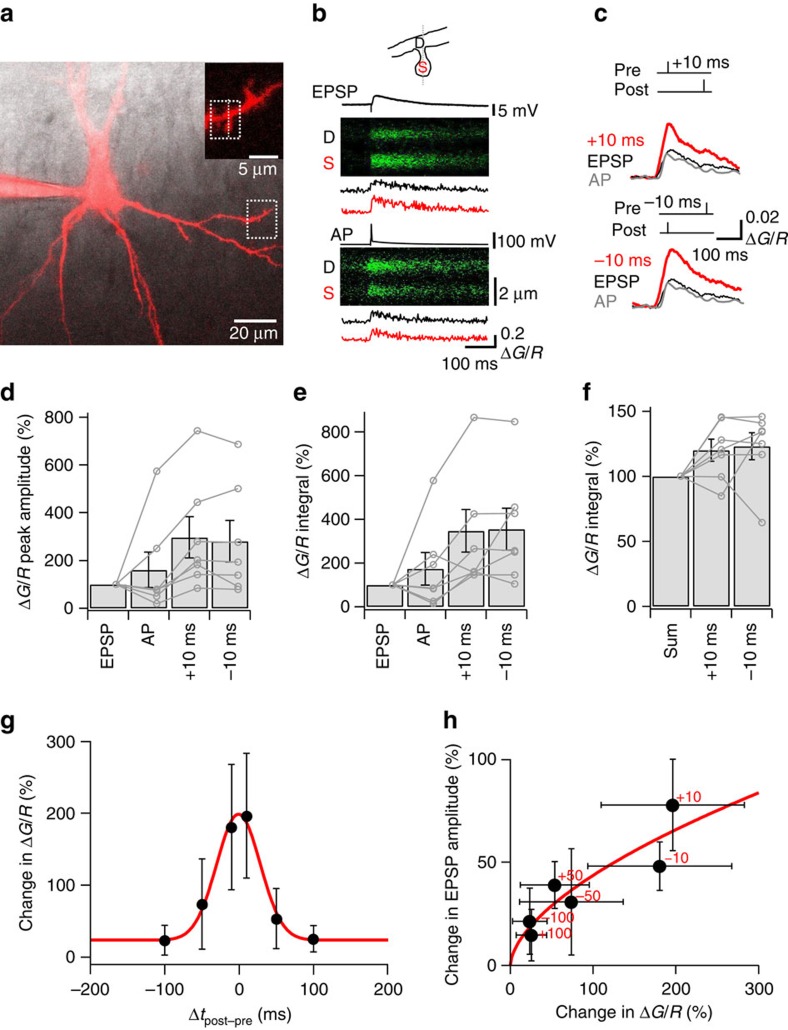
Summation of EPSP- and AP-induced [Ca^2+^] transients in spines of CA3 pyramidal neurons. (**a**) Fluorescence image of a CA3 pyramidal neuron loaded with Fluo-5F and Alexa Fluor 594 (maximum projection of stack of 48 1-μm confocal sections; excitation wavelength 594 nm), superimposed with the corresponding infrared-DIC image. The region indicated by the box is shown on an expanded scale in inset. (**b**) Spine [Ca^2+^] transients evoked by synaptic stimulation and backpropagated APs. Top, schematic illustration of the line scan configuration (dotted line, 400 Hz). Centre, [Ca^2+^] transient during a synaptically evoked EPSP. Bottom, [Ca^2+^] transient during a backpropagated AP. In each panel, upper graph represents membrane potential trace, middle shows *G* fluorescence signal against distance (vertical axis) and time (horizontal axis), and bottom indicates Δ*G*/*R* versus time in the dendrite (black) and the spine (red). (**c**) [Ca^2+^] transients during EPSPs (black traces), APs (grey traces), pre–postsynaptic pairing (Δ*t*=+10 ms; red trace, top), and post–presynaptic pairing (Δ*t*=−10 ms; red trace, bottom). Note that pairing markedly increased the peak amplitude of the [Ca^2+^] transient in comparison to isolated EPSPs. (**d**,**e**) Summary of the amplitude (**d**) and integral (**e**) of [Ca^2+^] transients evoked by single EPSPs, single APs, pre–postsynaptic pairing (Δ*t*=+10 ms) and post–presynaptic pairing (Δ*t*=−10 ms). Data were normalized to the EPSP value. Circles represent data from individual cells, bars indicate mean±s.e.m. Data from the same cell were connected by lines. (**f**) Supralinearity of summation. Summary bar graph shows integral values, normalized to the arithmetic sum of EPSP and AP values. Data in **d**–**f** from 7 cells. (**g**) A broad and symmetric [Ca^2+^] transient summation curve in CA3 pyramidal neuron spines. Peak amplitude of [Ca^2+^] transients during combined pre–postsynaptic or post–presynaptic stimulation, normalized to that of isolated EPSPs, was plotted against pairing time interval Δ*t* (7 cells total). Red curve, Gaussian function with offset fit to the data points (best-fit value for offset, 23.7%). Note that the [Ca^2+^] transient amplitude versus pairing interval curve was broad and symmetric, similar to the STDP curve. (**h**) Plot of change in EPSP amplitude against change of [Ca^2+^] transients during different pairing sequences. Numbers near symbols represent the values of Δ*t* between AP and EPSP (red, in ms). EPSP potentiation data were taken from Fig. 1. Red curve, power function fit to the data points (power coefficient 0.59).

To test whether the properties of spine [Ca^2+^] transients can explain the noncanonical STDP induction properties, we measured [Ca^2+^] transients in spines of CA3 pyramidal neurons, using the low-affinity Ca^2+^ indicator dye Fluo-5F (Fig. 2). Dendritic spines were located 50–200 μm from the soma, congruent with the location of CA3–CA3 synapses. [Ca^2+^] transients were evoked by either EPSPs following stimulation of nearby CA3 cell axons, or backpropagated APs following somatic current pulses (Fig. 2a,b). Control experiments with HFS (5 APs at 100 Hz) revealed that the Ca^2+^ indicator Fluo-5F was not saturated under our experimental conditions (Supplementary Fig. 5a). Synaptic stimulation and backpropagated APs evoked [Ca^2+^] transients with comparable amplitude, but different spatial profiles (Fig. 2b). [Ca^2+^] transients evoked by synaptic stimulation were larger in the spine than in the adjacent shaft, whereas transients evoked by backpropagated APs showed similar amplitudes in the two locations (Fig. 2b). [Ca^2+^] transients evoked by synaptic stimulation were enhanced by removal of Mg^2+^ from the bath solution and blocked by 20 μM 6-cyano-7-nitroquinoxaline-2,3-dione (CNQX) and 20 μM D-AP5, suggesting a contribution of NMDA-type glutamate receptors (Supplementary Fig. 5c,d). [Ca^2+^] transients evoked by backpropagated APs were completely blocked by 200 μM Cd^2+^ or 10 μM nimodipine, indicating that they were generated by Ca^2+^ inflow through voltage-gated Ca^2+^ channels (Supplementary Fig. 5b). [Ca^2+^] transients decayed with a time constant of 136±32 ms for synaptic stimulation and 145±25 ms for single backpropagated APs (9 and 13 cells, respectively), suggesting a broad window for temporal summation.

Pairing of EPSPs and backpropagated APs with short time intervals (Δ*t* either +10 ms or −10 ms) enhanced the amplitude of the [Ca^2+^] transients (Fig. 2c,d). Using the peak amplitude for quantification, [Ca^2+^] transients evoked by pre–postsynaptic sequences (that is, Δ*t*=+10 ms) were 2.96±0.87-fold larger than those by EPSPs alone (7 cells, *P*=0.016). Similarly, [Ca^2+^] transients evoked by post–presynaptic sequences (that is, Δ*t*=–10 ms) were 2.80±0.86-times larger than those by isolated EPSPs (7 cells, *P*=0.016; Fig. 2c,d). Similar results were obtained using the area under the [Ca^2+^] transients for quantification (Fig. 2e). [Ca^2+^] transients evoked by combined stimulation were significantly larger than the arithmetic sum of [Ca^2+^] transients evoked by APs or EPSPs in isolation. On average, the degree of nonlinearity, quantified as integral_EPSP+AP_/(integral_EPSP_+integral_AP_), was 120.0±8.5% for Δ*t*=+10 ms and 123.1±10.0% for Δ*t*=–10 ms (7 cells, *P*=0.014 in combined data set; Fig. 2f). Thus, pairing of EPSPs and APs led to a supralinear enhancement of spine [Ca^2+^] transients, potentially contributing to the associative nature of STDP (Fig. 1 and Supplementary Fig. 3).

To relate the summation properties of the [Ca^2+^] transient with the properties of STDP, we measured the amount of summation at different time intervals (Fig. 2d,e,g). Interestingly, the amplitude of the spine [Ca^2+^] transients was not significantly different between Δ*t*=+10 ms and Δ*t*=–10 ms (*P*=0.62; Fig. 2d,e). A plot of the summation of the [Ca^2+^] transient against pairing time interval Δ*t* revealed that the [Ca^2+^] transient summation curve was symmetric and broad (Fig. 2g). The half-duration of the curve was 69 ms (Fig. 2g), comparable to the STDP induction curve (Fig. 1i). These results were consistent with the idea that the temporally symmetric summation of [Ca^2+^] transients in dendritic spines may underlie the symmetric STDP induction rule in CA3–CA3 recurrent synapses. To further quantify the relation between the peak amplitude of [Ca^2+^] transients and the extent of potentiation, we plotted the two parameters against each other for all Δ*t* values (Fig. 2h). This analysis revealed a monotonically rising relation between potentiation and [Ca^2+^] transients, irrespective of the temporal order of APs and EPSPs.

### Dendritic signalling contributes to symmetric LTP induction

**Figure 3:**
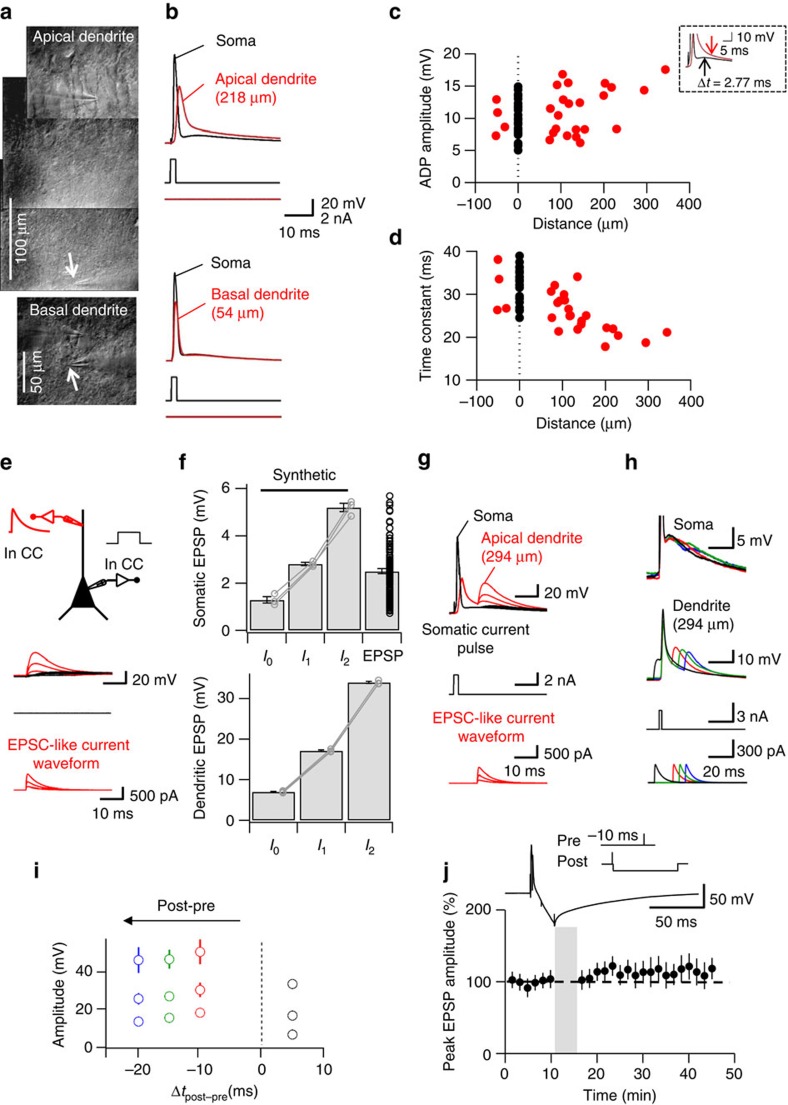
Dendritic afterdepolarization may represent the associative signal for LTP induction during post–presynaptic pairing. (**a**) Dendritic recording from apical (top) and basal CA3 pyramidal neuron dendrites (bottom). Infrared-DIC videomicrographs (photomontage for top graph) taken under experimental conditions. Arrows indicate dendritic recording sites. (**b**) The ADP has comparable amplitude at soma and dendrite. Dendritic recording sites 218 μm (top) and 54 μm (bottom) from the soma. Somatic (black) and dendritic AP (red), evoked by somatic current injection. (**c**) ADP amplitude remains constant as a function of distance from soma. Positive and negative distance values represent apical and basal recordings, respectively. Black, somatic ADP; red, dendritic ADP. Inset shows procedure to measure the ADP in the dendrite (at 294 μm). Note that the ADP amplitude shows a slight, but insignificant increase as a function of distance (26 simultaneous dendritic-somatic recordings, Spearman rank correlation coefficient *r*=0.26; *P*=0.065). (**d**) Similar plot as shown in **c**, but for ADP decay time constant. Note that the ADP becomes faster with increasing distance (*r*=0.72; *P*<0.001). (**e**) Properties of synthetic EPSPs, evoked by dendritic current injection. Top, schematic illustration of the recording configuration. Bottom, dendritic EPSPs (red) and somatic EPSPs (black). (**f**) Summary bar graph of peak amplitudes of synthetic EPSPs at the soma (top) and the dendrite (bottom). *I*_0_, *I*_1_ and *I*_2_ correspond to peak current amplitudes of 123, 306 and 610 pA, respectively (three simultaneous dendritic-somatic recordings). Right bar, top shows peak amplitudes of EPSPs evoked by extracellular stimulation (163 cells). (**g**) Summation of synthetic EPSPs with preceding ADPs. APs were evoked by somatic current injection, and synthetic EPSPs were generated by dendritic current injection (amplitudes *I*_0_, *I*_1_ and *I*_2_; Δ*t*=−10 ms). (**h**) Similar traces as shown in **g**, but for dendritic current injection amplitude *I*_1_ and Δ*t*=5 ms (black), –10 ms (red), –15 ms (green) and –20 ms (blue). (**i**) Plot of peak amplitude of the summated signal in the dendrite against AP–EPSP pairing interval Δ*t* (3 simultaneous dendritic-somatic recordings). (**j**) Conversion of the ADP into an afterhyperpolarization during a post–presynaptic sequence abolishes LTP. Top, post–presynaptic pairing protocol (Δ*t*=–10 ms) combined with hyperpolarizing somatic current injection and corresponding membrane potential trace. Bottom, plot of mean compound EPSP peak amplitude against experimental time before and after post–presynaptic pairing (Δ*t*=−10 ms) with afterhyperpolarization (9 cells).

Why are both the induction rule of LTP and the summation rule of spine [Ca^2+^] transients symmetric at CA3–CA3 synapses, in contrast to many other glutamatergic synapses^20^? A hallmark property of the excitability of CA3 pyramidal neurons is the marked afterdepolarization (ADP) following APs^27^,^28^,^29^. To test whether the ADP shapes the LTP induction rule, we performed recordings from the dendrites of CA3 pyramidal neurons (Fig. 3). In both somatic and dendritic recording sites, APs were followed by marked ADPs (Fig. 3b). The ADP was prominent in the dendrite, showing a slight, but not significant increase as a function of distance (26 simultaneous dendritic-somatic recordings, *P*=0.065; Fig. 3c). Furthermore, the ADP had a slow time course, with an average time constant of 34.2±1.1 ms at the soma 25.9±1.0 ms in the dendrite (26 dendritic-somatic recordings; Fig. 3d). In contrast, the AP peak amplitude declined as a function of distance and showed a faster time course (Fig. 3b)^21^. Thus, at distal locations, the ADP became an increasingly important component of the backpropagated signal. To test the summation of synaptic input with the ADP, we generated synthetic EPSPs by direct injection of exponential current waveforms into the dendrite (Fig. 3e). In these experiments, dendritic current injection was adjusted to mimic the peak amplitude of the somatic EPSP (Fig. 3f). When synthetic EPSPs followed the AP, EPSPs effectively summated with the ADP derived from the preceding spike (Fig. 3g). For realistic current injections, the peak dendritic depolarization evoked by pairing of AP and synthetic EPSP was ∼2-times larger than that of synthetic EPSPs in isolation (three simultaneous dendritic-somatic recordings; Fig. 3i).

If the ADP explains the potentiation with the post–presynaptic pairing sequence (Fig. 1d,e,i), abolishing the ADP should alter the extent of potentiation. To test this prediction, we combined our post–presynaptic induction protocol with a hyperpolarizing somatic current injection (Fig. 3j). Hyperpolarizing current pulses directly following the AP converted the ADP into an afterhyperpolarization (Fig. 3j, inset). Pairing this waveform with a subsequent EPSP (Δ*t*=–10 ms) failed to induce a significant potentiation in EPSP amplitude (9 cells, *P*=0.22; Fig. 3j). Furthermore, the amount of potentiation was significantly smaller than that induced by the corresponding pairing protocol lacking the hyperpolarizing current pulse (9 cells, *P*=0.03; Fig. 3j versus Fig. 1e; Table 1). Taken together, these results indicate that the ADP effectively propagated into the dendrites of CA3 pyramidal neurons, leading to nearly symmetric voltage changes during pre–postsynaptic and post–presynaptic sequences. Furthermore, conversion of the ADP into an afterhyperpolarization abolished potentiation obtained with the post–presynaptic sequence. Thus, the specific dendritic properties of CA3 pyramidal neurons^21^ may play a role in the generation of a broad, temporally symmetric STDP induction rule.

### Symmetric LTP induction facilitates storage and recall

**Figure 4:**
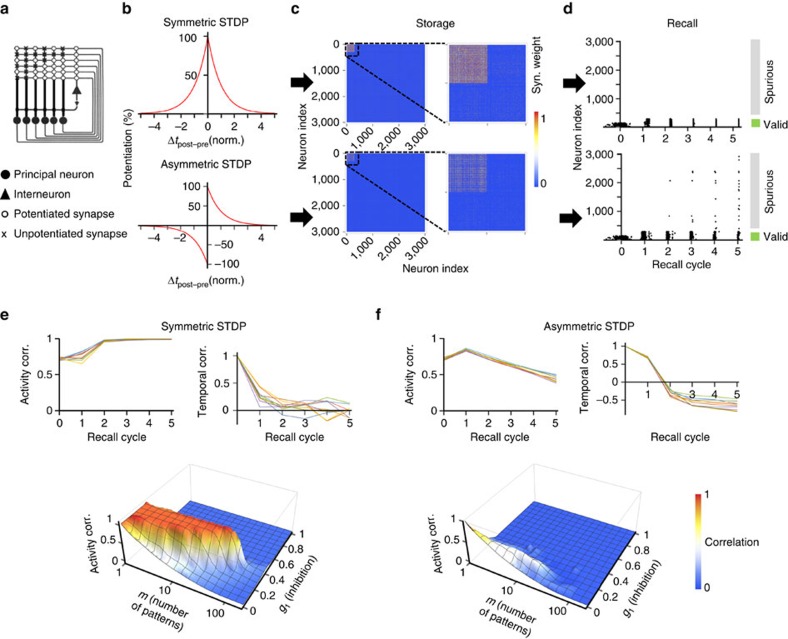
Symmetric STDP facilitates storage and retrieval of patterns in an autoassociative network model. (**a**) Schematic illustration of network topology. The model is composed of several principal neurons (filled circles) and an inhibitory interneuron (filled triangle). Principal cells are interconnected by excitatory synapses (potentiated, open circles; unpotentiated, crosses). In the schematic, there are six pyramidal cells; the real model was composed of 3,000 pyramidal neurons. The mixture of potentiated and unpotentiated synapses in the matrix was generated by prior application of three binary activity patterns (001011, 101010 and 000111). Modified from McNaughton and Morris^4^. See Supplementary Table 1. (**b**) Plasticity rules. Top, symmetric STDP rule, as supported by the present results. Bottom, asymmetric STDP rule^15^,^16^,^17^. Δ*t* was given in normalized units, which could correspond to one theta oscillation cycle (∼200 ms). (**c**) Storage of patterns in the synaptic matrix. Synaptic weight was represented as temperature map (red, maximal potentiation; blue, unpotentiated). A single test pattern in the first 300 neurons and 10 additional patterns in randomly selected neurons were applied during storage, with randomized spike time in both cases. Ordinate and abscissa represent index of pre- and postsynaptic neuron, respectively. Insets (right) show expanded views for first 600 cells. Note that the symmetric STDP rule (top) induces a higher average potentiation than the asymmetric rule (bottom). (**d**) Recall of patterns in the network model. Recall was triggered by partial test patterns (50% valid firings, no spurious firings in comparison to original pattern) with randomized spike timing. With the symmetric STDP rule (top; proportionality factor of inhibition *g*_1_=0.3), the original pattern was perfectly retrieved after three recall cycles. With the asymmetric rule (bottom; *g*_1_=0.1), retrieval was only partial, with decrease in valid and increase in spurious firing. (**e**,**f**) Top, dependence of activity correlation (left) and spike-time correlation for active cells (right) on recall cycle number for symmetric (top) and asymmetric STDP rules (bottom). Coloured lines represent pattern correlation trajectories for 10 patterns. For the symmetric STDP rule, activity correlation increases, whereas spike-time correlation is eliminated. For the asymmetric STDP rule, activity correlation declines, whereas spike-time correlation becomes inverted. Bottom, three-dimensional plot of activity correlation at the 5^th^ recall cycle versus pattern load *m* and proportionality factor of inhibition *g*_1_ for symmetric (**e**) and asymmetric (**f**) STDP rules. For the symmetric rule, capacity was 58.1, whereas for the asymmetric rule it was 4.5. Corr., correlation.

What are the implications of noncanonical STDP induction rules for the memory function of the CA3 network? To address this question, we simulated storage and recall in a network model of pattern completion (Fig. 4). The implementation followed previous models^2^,^3^,^12^,^13^ (Fig. 4a), but additionally incorporated the time dependence of both spiking and plasticity (Methods and Supplementary Table 1). 3,000 integrate-and-fire type neurons were connected by excitatory synapses represented as delta-function current sources. Excitatory synapses were endowed with either symmetric (this paper) or asymmetric plasticity rules^15^,^16^,^17^ (Fig. 4b). In the storage phase, a defined test pattern with temporal spread of activity was applied to the first 300 cells (corresponding to a proportional activity level of 0.1=300/3,000; Supplementary Table 1). Subsequently, several additional random patterns were applied, leading to potentiation of synapses in the synaptic matrix (Fig. 4c). In the recall phase, an incomplete test pattern was employed (Fig. 4d). For perfect recall, the original test pattern (that is, the first 300 cells) should be selectively reactivated after a number of recall cycles. By contrast, for impaired recall, the number of valid firings would be reduced, whereas the number of spurious firings is expected to increase. Finally, we quantified the quality of recall as the correlation between original and retrieved patterns, and capacity of the network as the maximal number of patterns that could be loaded without recall impairment (Methods and Supplementary Table 1).

When our new plasticity rule was implemented, the recall of the original patterns was robust, and the capacity of the network was 58.1 patterns (Fig. 4e). In contrast, when an asymmetric STDP rule^17^ was incorporated, the recall of the pattern was markedly impaired (capacity 4.5 patterns; Fig. 4f). Thus, in our model, the symmetric STDP induction rule facilitated the storage and recall of information by incomplete input, conveying the ability of pattern completion. Remarkably, the symmetric STDP rule led to only minimal temporal correlation between spike times in the original patterns and the retrieved patterns, because synchronization of activity emerged during the recall phase (Fig. 4e, top right). In contrast, the asymmetric STDP rule generated a significant negative temporal correlation between original and retrieved patterns after the first five recall cycles (Fig. 4f, top right). Intuitively, the cells that generate APs last during the storage phase show maximal potentiation of input synapses, and therefore fire first during recall. Thus, the symmetric STDP rule is advantageous for pattern completion, whereas asymmetric rules may be superior for the storage and recall of temporal AP sequences, for example, during spatial learning^30^.

## Discussion

The present paper identifies a new STDP induction rule at CA3–CA3 recurrent synapses. In contrast to previously reported induction rules^20^, the STDP curve at CA3–CA3 synapses was broad and symmetric. Although anti-Hebbian STDP induction rules were previously reported^25^,^31^,^32^,^33^,^34^,^35^,^36^, none of the prior studies demonstrated a broad and symmetric potentiation curve. Both blockers of NMDA receptors and L-type Ca^2+^ channels blocked STDP, showing that two independent Ca^2+^ sources were required for plasticity induction at these synapses^15^,^37^. Spine [Ca^2+^] transients mediated by NMDA receptors and L-type Ca^2+^ channels showed temporally symmetric summation. Thus, the properties of spine Ca^2+^ signalling provide a possible mechanistic explanation for the temporally symmetric plasticity rule.

Backpropagated dendritic APs are key associative signals in STDP induction^38^,^39^. In a pre–postsynaptic activity sequence, backpropagated APs are thought to relieve the Mg^2+^ block of the NMDA receptor^40^. This mechanism may also operate in CA3–CA3 synapses, and may be particularly effective, because of active AP backpropagation caused by the high dendritic Na^+^ channel density in these cells^21^. But what are the dendritic mechanisms of post–presynaptic pairing? Our results suggest that the ADP^27^,^28^,^29^, a hallmark of CA3 pyramidal neuron excitability, may play a critical role. Two properties of the ADP make it particularly suitable as an associative signal. First, the ADP propagates into dendrites without attenuation, and second, it effectively summates with subsequent EPSPs. Thus, in CA3 cells post–presynaptic sequences can produce large dendritic depolarizations, which will be particularly effective in both relief of Mg^2+^ block of NMDA receptors and activation of voltage-gated Ca^2+^ channels. These summation properties appear to be specific for CA3 pyramidal neurons, as in layer 5 neocortical cells APs shunt subsequent EPSPs, rather than boosting their amplitude^41^,^42^. Previous studies in CA3 pyramidal neurons in organotypic slice culture demonstrated STDP with asymmetric induction rules^9^. The apparent difference may be caused by developmental factors^43^, as our results were obtained in relatively mature animals (postnatal day 21–29), whereas cultured cells are likely to be less developed. Consistent with this idea, post–presynaptic pairing induced LTD at CA3–CA3 recurrent synapses in acute slices from young animals (R.K.M., unpublished observations). Alternatively, the difference may be caused by costimulation of neuromodulatory inputs in extracellular stimulation experiments. Recent results suggested the involvement of dopamine receptors in the regulation of STDP induction rules^44^. However, post–presynaptic activity sequences induced LTP in the presence of dopamine receptor antagonists (R.K.M., unpublished observations). Furthermore, post–presynaptic activity sequences induce LTD in Schaffer collateral–CA1 pyramidal neuron synapses^18^.

If STDP induction at CA3–CA3 synapses is symmetric, and an LTD component is lacking, how is stability achieved in the network? Several additional regulatory mechanisms may be at work. For example, homeostatic plasticity at CA3–CA3 synapses may counteract saturation in the network^45^,^46^. In addition, homeostatic plasticity at mossy fibre synapses on CA3 pyramidal neurons could play a role^47^. Finally, LTD has been reported in hippocampal mossy fibre–CA3 pyramidal neuron synapses early in development^48^,^49^. Whether and how such LTD mechanisms can be reactivated in the mature brain remains to be determined.

Our autoassociative network model shows that the novel STDP rule enhances the computational power of the network, increasing the reliability of pattern completion and the storage capacity^2^. The temporally symmetric STDP rule addresses two issues with previous models. First, it solves the problem that synchronous activity in ensembles generates LTD via a post–presynaptic sequence, because the time for dendritic propagation is shorter than that for axonal propagation of the AP^21^. Second, it addresses how autoassociative network models can work under conditions of slightly asynchronous activity in CA3 pyramidal neuron ensembles. In particular, the broad and temporally symmetric STDP rule could be useful for the reactivation of cell assembly patterns of freely moving animals in open fields^50^, and for the incorporation of contralateral CA3 pyramidal neurons into bi-hemispheric neuronal assemblies.

## Methods

### Slice preparation and patch-clamp recording

Transverse hippocampal slices (thickness, 350 μm) were prepared from the brains of 21- to 29-day-old Wistar rats of either sex^21^. Animals were lightly anaesthetized using isoflurane (Forane; Abbott) and killed by rapid decapitation. Experiments were performed in strict accordance with institutional, national and European guidelines for animal experimentation and were approved by the Bundesministerium für Wissenschaft, Forschung und Wirtschaft (A. Haslinger, Vienna). Slices were cut in ice-cold sucrose-containing physiological saline using a vibratome (VT1200, Leica Microsystems), incubated in a maintenance chamber filled with sucrose-saline at ∼36 °C for ∼45 min, and subsequently stored at room temperature. Slices were then individually transferred into a recording chamber perfused with standard physiological saline. Recordings were performed at room temperature (∼22 °C, range: 21–23 °C) or near-physiological temperature (∼33 °C; range 32–34 °C; Supplementary Fig. 4). In the experiments at ∼33 °C, temperature was controlled using a water jacket or a temperature controller (Sigmann Elektronik). Slices were used for maximally 6 h after dissection.

Patch pipettes were pulled from thick-walled borosilicate glass tubing (outer diameter: 2 mm, inner diameter: 1 mm) using a horizontal pipette puller (P-97, Sutter Instruments). When filled with internal solution, the open-tip resistance was 4–6 MΩ. All measurements were performed with an Axoclamp 700B amplifier (Molecular Devices). Signals were low-pass filtered at 5–10 kHz and digitized at a sampling rate of 20 kHz with a CED 1401 plus interface (Cambridge Electronic Design). Pulse protocols were generated using custom-made data acquisition software (FPulse 3.33; U. Fröbe, Freiburg) running under Igor Pro 6.22 (WaveMetrics). Whole-cell patch-clamp recordings were made from the soma of visually identified pyramidal neurons located in the *stratum pyramidale* of the CA3b subfield (Fig. 1a). Resting membrane potential was measured immediately after membrane rupture.

EPSPs were recorded in the presence of 10 μM SR-95531 (gabazine). To stimulate input synapses, a borosilicate glass pipette (2–3 MΩ) filled with 1 M NaCl was placed in *stratum oriens* (∼200 μm away from the soma of the recorded CA3 pyramidal cell). As axons of CA3 pyramidal neurons often traverse the pyramidal cell layer, this location is expected to activate synapses on both apical and basal dendrites of target cells^7^,^22^, but avoids stimulation of mossy fibre inputs. Axons were stimulated with brief voltage pulses (3–25 V amplitude, 100 μs duration) via a stimulus isolation unit at a basal frequency of 0.1–0.2 Hz. Stimulus intensity was chosen to give evoked EPSP amplitude of 1–5 mV (2.50±0.11 mV; 163 cells), corresponding to ∼2–10 unitary synaptic inputs^23^,^51^.

To verify the selective stimulation of CA3–CA3 recurrent synapses, the effect of bath application of (2*S*,2'*R*,3'*R*)-2-(2',3'-dicarboxycyclopropyl)glycine (DCG-4; 1 μM) was tested at the end of the experiment in a subset of recordings. In the tested cells, average inhibition by DCG-4 was 98.7±2.5% (range: 83–116%; 62 cells), making a contamination by mossy fibre synapses unlikely^52^. To confirm the identity of the recorded neurons, a subset of cells was filled with biocytin during recording and labelled using 3,3′-diaminobenzidine as chromogen^21^ (20 cells), and another subset was filled with Alexa Fluor 594 and examined in confocal stacks (22 cells). All labelled neurons showed the characteristic morphological properties of CA3 pyramidal neurons, including a high density of spines on their dendritic branches.

LTP was induced with either pairing or HFS paradigms. The pairing protocol consisted of 300 repetitions of a single presynaptic stimulation paired with a postsynaptic AP at different time intervals at 1 Hz. Single APs were evoked by brief current injections (4 nA amplitude, 2 ms duration) to the soma. To ensure the absence of multiple spiking during induction, AP–EPSP sequences were carefully monitored; in none of the STDP experiments, multiple spiking occurred during the induction period. The HFS protocol consisted of 4 trains of 100 stimuli at 100 Hz delivered every 10 s.

For EPSP recording, series resistance was fully compensated, and pipette capacitance was ∼70% compensated. Both resting membrane potential and input resistance (*R*_in_) of the recorded CA3 pyramidal neuron were monitored over experimental time. Experiments were discarded if the resting membrane potential depolarized above −60 mV, *R*_in_ fell below 100 MΩ, or *R*_in_ changed by more than 30% during the recording.

### Spine Ca^2+^ imaging

Imaging of CA3 pyramidal neuron spines was performed using an upright microscope (DM 6000 FS, Leica Microsystems) equipped with a confocal laser scanhead (TCS SP5 II, Leica Microsystems) using either a × 20 (numerical aperture (NA)=1.0) or a × 63 (NA=0.9) water immersion objective. Before imaging, neurons in the CA3b area were identified with infrared-differential interference contrast (infrared-DIC) videomicroscopy using a CCD camera (DFC365 FC, Leica Microsystems). CA3 pyramidal neurons were loaded with 100 μM of the Ca^2+^ indicator dye Fluo-5F and 50 μM of the Ca^2+^-insensitive dye Alexa Fluor 594 (both Invitrogen) via the patch pipette^53^. To allow for a proper equilibration of the dyes, fluorescence signals were measured 40–50 min after break-in, and series resistance was kept below 25 MΩ. Excitation wavelength was 488 nm (argon laser) for Fluo-5F and 561 nm for Alexa Fluor 594 (diode pumped solid state laser).

To detect spines that responded to the stimulation of CA3–CA3 recurrent synapses, we scanned a dendritic area of 11.5 × 11.5 μm^2^ with 10 frames (128 × 128 pixels) acquired at a rate of 3.9 Hz while a stimulation electrode was placed in the vicinity of a dendrite (typically at <20 μm distance). Reactive spines were identified by an increase in the green fluorescence in the presence of a modified Mg^2+^-free physiological saline. In total, < 5% of spines were reactive. For subsequent recording, standard physiological saline was used.

For imaging, regions of interest were set to basal dendrites 50–200 μm from the soma. Line scans of spines, dendrites or both were acquired every 2.5 ms. Distance was measured as the shortest linear path along the dendrite from the scanning site to the tip of the somatic pipette. Fluorescent transients were recorded in response to extracellular synaptic stimulation or backpropagated APs evoked by brief somatic current pulses (4 nA, 2 ms), both delivered every 15 s. All recordings were made in the presence of 10 μM gabazine in the bath solution. Intracellular [Ca^2+^] transients were analysed with custom-made routines implemented in Fiji^54^. Fluorescence was expressed as green over red ratio, Δ*G*(*t*)/*R*, where *G*(*t*) is the fluorescence in the green channel for each time point and *R* is the mean fluorescence signal in the red channel over each recording epoch^37^,^53^. Baseline fluorescence was measured in a 50-ms time window before stimulation, and peak fluorescence was determined in a ±10-ms time window around the absolute maximum. Care was taken to avoid dye ejection from the patch pipette during cell approach and sealing procedure. As background was minimal, no correction was performed. [Ca^2+^] transients shown in figures represent average fluorescence of 5–10 consecutive line scans, unless noted differently. The average decay time constant of the [Ca^2+^] transients was ∼150 ms, consistent with a relatively low affinity of Fluo-5F^53^.

### Dendritic recording

Dendritic recordings from CA3 pyramidal neurons were obtained as previously described^21^. First, a somatic recording configuration was obtained, using an internal solution containing Alexa Fluor 488 (100 μM, Invitrogen). Second, after ∼10 min of somatic whole-cell recording, fluorescently labelled dendrites were traced from the CA3 pyramidal neuron soma into dendritic layers using a Nipkow spinning disk confocal microscope (Ultraview live cell imager, Perkin Elmer, equipped with an Orca camera, Hamamatsu, and an argon/krypton gas laser or a solid-state laser; excitation wavelength 488 nm). Exposure times were minimized to avoid phototoxicity. Finally, fluorescent and infrared-DIC images were compared and CA3 pyramidal neuron dendrites were patched under infrared-DIC. For dendritic recordings, patch pipettes had resistances of 8–23 MΩ. For subsequent analysis, only recordings with series resistance ≤60 MΩ were used. Recordings were made from both apical and basal dendrites of CA3 pyramidal cells.

### Solutions and chemicals

Sucrose-based solution for dissection and storage of slices contained 87/64 mM NaCl, 25 mM NaHCO_3_, 2.5 mM KCl, 1.25 mM NaH_2_PO_4_, 7 mM MgCl_2_, 0.5 mM CaCl_2_, 25 mM glucose and 75/120 mM sucrose. Physiological saline for experiments (artificial cerebrospinal fluid) contained 125 mM NaCl, 25 mM NaHCO_3_, 2.5 mM KCl, 1.25 mM NaH_2_PO_4_, 1 mM MgCl_2_, 2 mM CaCl_2_ and 25 mM glucose. Slices were superfused at a rate of 2.5–5.0 ml min^−1^ (recording chamber volume ∼2 ml). For current-clamp recording, intracellular solution was composed of 140 mM K-gluconate, 20 mM KCl, 10 mM HEPES, 0.1 mM EGTA, 2 mM MgCl_2_, 4 mM Na_2_ATP and 0.3 mM NaGTP, pH adjusted to 7.28 with KOH (∼300 mOsm). For imaging, intracellular solution contained 135 mM K-gluconate, 20 mM KCl, 10 mM HEPES, 2 mM MgCl_2_, 4 mM Na_2_ATP, 0.3 mM NaGTP, 2 mM ascorbic acid, 50 μM Alexa Fluor 594 and 100 μM Fluo-5F, pH adjusted to 7.28 with KOH (∼300 mOsm). In subsets of experiments, 10 mM phosphocreatine was included. Chemicals were from Merck, except sucrose, MgCl_2_, EGTA, HEPES, K-gluconate, ascorbic acid and phosphocreatine (Sigma-Aldrich), as well as Alexa Fluor 594 and Fluo-5F (Life Technologies).

Extracellularly applied chemicals were kept in concentrated stock solution in ultrapure water at –20 °C and dissolved in physiological saline immediately before the experiment. These included: D-AP5 (Biotrend), nimodipine (3-(2-methoxyethyl) 5-propan-2-yl 2,6-dimethyl-4-(3-nitrophenyl)-1,4-dihydropyridine-3,5-dicarboxylate; Tocris), DCG-4 (Tocris) and SR-95531 (2-(3-carboxypropyl)-3-amino-6-(4 methoxyphenyl)pyridazinium bromide; gabazine; Biotrend). Intracellularly applied EGTA and QX-314 (*N*-(2,6-dimethylphenylcarbamoylmethyl) triethylammonium chloride; Biotrend) were directly added to the pipette solution; both substances were allowed to diffuse into the recorded cell for ∼20 min before the experiment was started.

### Data analysis

Analysis of evoked EPSPs was performed with Stimfit (version 0.13 or 0.14) (ref. 55) or equivalent custom-made routines written in C or Python 2.6 or 2.7. The rise time of the EPSPs was determined as the time interval between the points corresponding to 20% and 80% of the peak amplitude, respectively. The peak amplitude was determined as the mean or maximum within a window of 1 or 2 ms duration, respectively, following the stimulus. The synaptic latency was determined as the time interval between the centre of the stimulus artefact and the onset of the subsequent EPSP; the onset point was determined from the intersection of a line through the 20 and 80% points with the baseline. The decay phase of the EPSPs was fit with a monoexponential function using a nonlinear least-squares fit algorithm. To assure reliable quantification of LTP, only recordings with stationary baseline, as tested by a Spearman rank-correlation test (*P*>0.05), were included. The magnitude of LTP was quantified by ratio between the mean EPSP peak amplitude 20−30 min after the induction paradigm and the mean value in a 10-min time interval before induction. The amplitude of the ADP in somatodendritic recordings was measured from resting potential. At the somatic recording site, the peak amplitude of the ADP was measured directly. For measuring the ADP amplitude at the dendrite, the time difference *δ* between the 50% decay time point of the AP and the peak of the ADP was first quantified at the soma. Next, the 50% decay time point of the backpropagated AP (*t*_50, dendrite_) was determined in the dendrite. Finally, the amplitude of the dendritic ADP was measured at the time point *t*_50, dendrite_+*δ*.

### Storage and recall in autoassociative network models

Simulations of pattern completion in autoassociative network models were performed following previous work^2^,^3^,^12^,^13^ (Supplementary Table 1). 3,000 integrate-and-fire type neurons^56^ were connected by excitatory synapses with a probability of 0.5. Excitatory synapses were represented as delta-function current sources. For each storage cycle (for example, a theta cycle of 200 ms duration), synaptic plasticity was implemented using different STDP rules based on piecewise exponential functions. In the symmetric rule (Fig. 4b, top), the potentiation function was *y*(Δ*t*)=Exp[-Abs(Δ*t*)/*τ*_pot_], where Δ*t* is the time difference between post- and presynaptic spike, *τ*_pot_ is the time constant and Abs is the absolute value. *τ*_pot_ was set to one time unit, corresponding to one storage cycle. In the asymmetric rule^15^,^16^,^17^ (Fig. 4b, bottom), the potentiation function was *y*(Δ*t*)=Sign(Δ*t*) × Exp[-Abs(Δ*t*)/*τ*_pot_], where Sign is the signum function. Basal synaptic strength (*j*_0_) was assumed as 0. The total activity level *a* was set to 0.1. Activity in the network was assumed to show normally distributed spike times, with a standard deviation *σ*_t_ of 0.2 time units, which would correspond to a 20%-fraction of a storage cycle. The lower and upper boundaries for synaptic efficacy were 0 and 1, respectively.

Neuronal activity during progressive recall was simulated during 5–10 time steps. Excitatory synaptic potentials in the network were generated according to the function *v*(*t, δ*)=Step(*t*-*δ*) × Exp[-(*t*–*δ*)/*τ*_m_], where *δ* is a delay determined by the spike time of the presynaptic neuron, and *τ*_m_ is the synaptic potential decay time constant (1 time unit, corresponding to one recall cycle, for example, a theta, gamma or ripple cycle). For each recall cycle, the total input to the *i*^th^ neuron at time *t* was calculated as 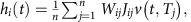
where *W* denotes the connectivity matrix, *J* represents synaptic weight matrix, *T*_*j*_ is the spike time of the *j*th presynaptic neuron in the previous recall cycle (–infinity for silent neurons) and *n* is the number of neurons. A neuron was assumed to fire APs in a given recall cycle if the condition 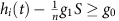 was met, where *S* is the total activity in the network in the prior cycle, *g*_0_ is the firing threshold (set to 0) and *g*_1_ is the proportionality factor of inhibition (varied between 0 and 1; Supplementary Table 1)^12^,^13^. The spike time was calculated by solving the equation 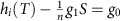. Retrieval was tested with incomplete random patterns, in which the proportion of valid firing neurons was 0.5 (that is, *b*_1_=0.5; Supplementary Table 1)^12^,^13^, and the proportion of spuriously firing neurons was 0 (that is, *b*_n_=1; Supplementary Table 1)^12^,^13^. The overlap between original and recalled patterns was computed as the correlation coefficient between original and final activity vectors. The absolute capacity of the network was defined as the maximum of the product function of pattern correlation times pattern load. The capacity of the implemented 3,000-neuron network was up to 100 patterns, but was substantially increased for real-sized 330,000-neuron networks^13^ (P.J., unpublished observations). Simulations were implemented in Mathematica, Matlab, C or C++, and run on PCs or a scientific computer cluster (Supermicro) using GNU/Debian Linux (x86_64), a GNU C compiler (GCC, 4.9.2) and the GNU scientific library (GSL, 1.16). Computer code will be provided upon request.

### Statistics and conventions

All values are given as mean±s.e.m. Error bars in the figures also indicate s.e.m. (shown only if larger than symbol size). As data points often appeared to be non-normally distributed, nonparametric were preferred over parametric statistical tests. Statistical significance was tested using a Kruskal–Wallis test, a two-sided Wilcoxon signed rank test for paired data, or a two-sided Wilcoxon rank sum test for unpaired data (Igor Pro 6.32). In case of multiple comparisons, Bonferroni correction was performed. Differences with *P*<0.05 were considered significant. Throughout the figures, *P<0.05, **P<0.01 and ***P<0.001. Membrane potential values were specified without correction for liquid junction potentials. Data included in this paper are based on recordings from a total of 211 CA3 pyramidal neurons (163 for EPSP recording, 22 for Ca^2+^ imaging and 26 for dendritic recording).

## Additional information

**How to cite this article:** Mishra, R. K. *et al*. Symmetric spike timing-dependent plasticity at CA3–CA3 synapses optimizes storage and recall in autoassociative networks. *Nat. Commun.* 7:11552 doi: 10.1038/ncomms11552 (2016).

## Supplementary Material

Supplementary Information Supplementary Figures 1-5, Supplementary Table 1 and Supplementary References.

## Figures and Tables

**Table 1:** Synaptic plasticity at CA3–CA3 recurrent synapses.

Basic properties of compound EPSPs at CA3–CA3 recurrent synapses (*n*=42)	Value
Peak EPSP (mV)	2.5±0.2
Latency (ms)	2.7±0.1
20–80% Rise time (ms)	4.9±0.2
Decay time constant (ms)	103.7±4.2

Condition	Value (% of baseline)	Number of cells, *n*	*P*
LTP induced by HFS*			
HFS control	196.8±18.4	14	0.0002†
HFS+D-AP5 (20 μM, bath application)	98.7±11.0	6	0.0007, 0.002‡
HFS+EGTA (20 mM, intracellular)	102.4±11.2	5	0.0007, 0.006‡
HFS+nimodipine (10 μM, bath application)	82.0±8.0	5	0.0007, 0.001‡
			
*LTP induced by pairing of pre- and postsynaptic activity*§			
STDP (+10 ms) control	178.0±22.1	9	0.004†
STDP (+10 ms) D-AP5 (20 μM, bath application)	101.2±5.2	7	0.0007, 0.03‡
STDP (+10 ms) EGTA (20 mM, intracellular)	101.8±7.2	8	0.0007, 0.02‡
STDP (+10 ms) nimodipine (10 μM, bath application)	91.1±6.0	5	0.0007, 0.04‡
STDP (–10 ms) control	159.3±11.5	17	0.00002†
STDP (–10 ms) D-AP5 (20 μM, bath application)	100.5±4.2	6	0.0005, 0.001‡
STDP (–10 ms) EGTA (20 mM, intracellular)	103.0±9.0	6	0.0005, 0.01‡
STDP (–10 ms) nimodipine (10 μM, bath application)	105.0±6.1	5	0.0005, 0.01‡
Presynaptic stimulation only	102.8±2.9	6	0.31†
Postsynaptic APs only	95.7±4.4	6	0.38†
STDP (–10 ms) with hyperpolarizing current pulse	114.6±13.0	9	0.03||, 0.22†

AP, action potential; D-AP5, D-2-amino-5-phosphonovaleric acid; EGTA, ethylene glycol-bis(2-aminoethylether)-*N*,*N*,*N*′,*N*′-tetraacetic acid; HFS, high-frequency stimulation; EPSP, excitatory postsynaptic potential; LTP, long-term potentiation; STDP, spike timing-dependent plasticity.

All data presented in table were obtained at room temperature. For details, see the text.

^*^The HFS protocol consisted of 4 trains of 100 stimuli at 100 Hz delivered every 10 s.

^†^Significance was tested using Wilcoxon signed rank test (control versus test period after induction).

^‡^Significance was assessed using Kruskal–Wallis test, followed by Wilcoxon–Mann–Whitney two-sample rank test (control versus experimental manipulation), using Bonferroni correction for multiple comparisons.

^§^The pairing protocol consisted of 300 repetitions of a single presynaptic stimulation paired with a postsynaptic AP at different time intervals at 1 Hz.

^||^Significance was assessed using Wilcoxon–Mann–Whitney two-sample rank test (control versus experimental manipulation).
