# Supplementary material for: Symmetric spike timing-dependent plasticity at CA3–CA3 synapses optimizes storage and recall in autoassociative networks
Source: Nat Commun. 2016 May 13;7:11552. doi: 10.1038/ncomms11552 (PMC4869174; doi:10.1038/ncomms11552)
Supplement: Supplementary Information — Supplementary Figures 1-5, Supplementary Table 1 and Supplementary References. [file ncomms11552-s1.pdf]

Supplementary Figure 1. Basic properties of compound EPSPs at hippocampal CA3–CA3 cell synapses.

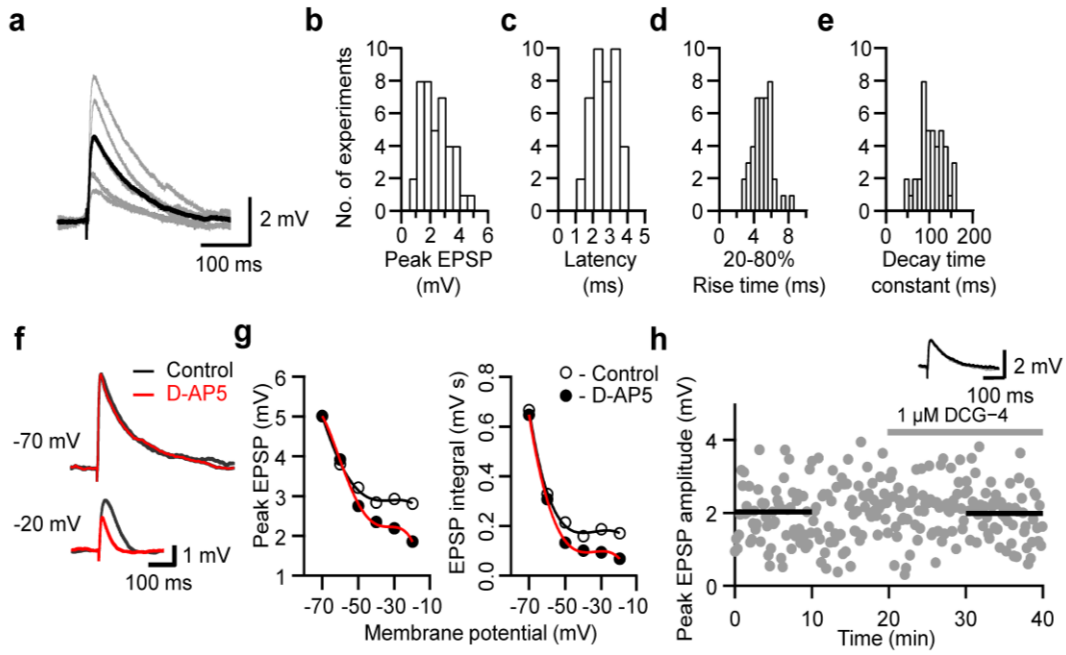

(a) EPSPs were evoked by extracellular stimulation of the recurrent collaterals and pharmacologically isolated in the presence of 10 µM gabazine. Individual EPSPs are shown in gray and the average is shown in black.

(b–e) Distributions of mean peak amplitude (**b**), latency (**c**), 20–80% rise time **(d)** and decay time constant of EPSPs (**e**, 42 cells).

(f) EPSP traces in control conditions (black) and in D-AP5 (red) at –70 mV (top) and –20 mV (bottom). 5 mM QX-314 was added to the pipette solution to prevent AP initiation in the postsynaptic cell.

(g) Depolarization uncovers an NMDA receptor-mediated component in the EPSP. Left, EPSP peak amplitude; right, EPSP integral, both plotted against membrane potential. Open symbols, data in control; filled symbols, data in the

presence of 20 µM D-AP5. Red curves represent 5th order polynomial functions fit to the data points. Note the presence of a D-AP5-sensitive component at membrane potentials > –50 mV. Same experiment as shown in **f**.

(h) Evoked EPSPs were insensitive to bath application of 1 µM DCG-4, suggesting that they originated at CA3–CA3 recurrent synapses. Data from a representative experiment; horizontal gray bar indicates DCG-4 application time. Note that DCG-4 had only minimal effects on EPSP amplitude, implying the absence of contamination from mossy fiber synapses (Kamiya et al., 1996). Inset shows overlay of EPSP traces in control conditions and in the presence of DCG-4 (black and gray, respectively).

Supplementary Figure 2. LTP at hippocampal CA3–CA3 cell synapses induced by a high-frequency stimulation (HFS) protocol.

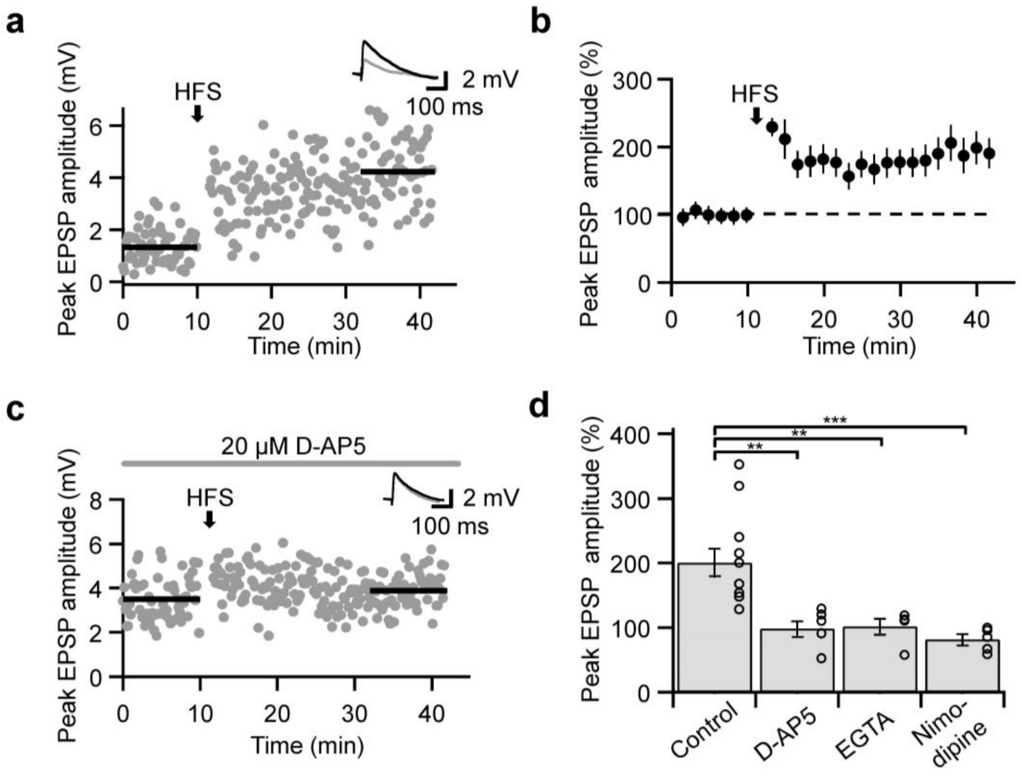

(a) HFS induced robust LTP at recurrent CA3–CA3 synapses. Plot of compound EPSP peak amplitude against experimental time before and after application of HFS (arrow) in a single experiment. Inset shows the average of 60 EPSP responses before (gray) and 30 min after HFS (black).

(b) Plot of average compound EPSP peak amplitude against experimental time before and after application of HFS (arrow) in 14 experiments. EPSP amplitude was normalized to the control value before HFS (dashed line).

(c) Bath application of 20 µM of the NMDA receptor antagonist D-AP5 prevented the increase of EPSP amplitude after HFS, showing that HFS-

induced LTP induction required the activation of NMDA receptors. Single representative experiment.

(d) Multiple Ca^{2+} sources were necessary for HFS-induced LTP. Summary bar graph showing the effect of the NMDAR antagonist D-AP5 (extracellular, 20 µM; 6 cells), the Ca^{2+} chelator EGTA (intracellular, 20 mM; 5 cells), and the L-type Ca^{2+} channel blocker nimodipine (extracellular, 10 µM; 5 cells). LTP with both pairing paradigms was largely abolished by all manipulations.

Supplementary Figure 3. Pairing-induced LTP at CA3–CA3 synapses is associative, requiring joint pre- and postsynaptic activity.

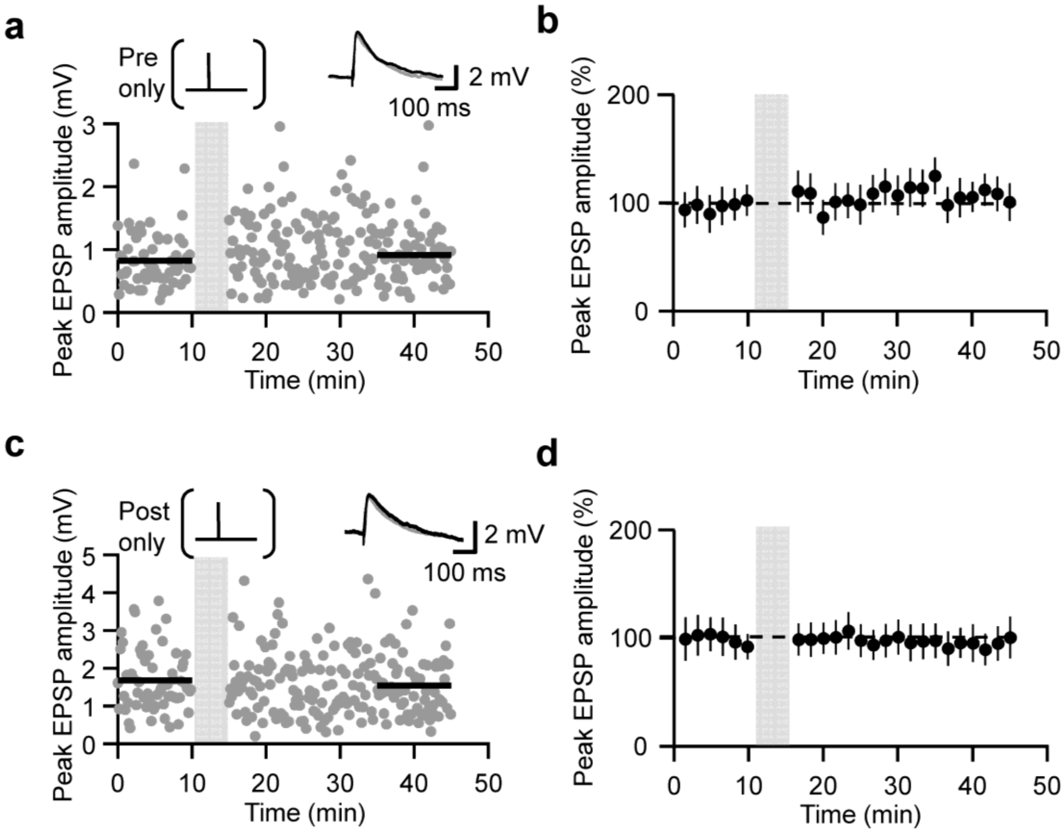

(a, b) Isolated presynaptic stimulation fails to induce LTP. Plot of average compound EPSP peak amplitude against experimental time before and after application of isolated presynaptic stimulation. Data from a single representative experiment (**a**) and mean from 6 experiments (**b**).

(c, d) Isolated postsynaptic stimulation fails to induce LTP. Similar plot as in **a, b**, but for postsynaptic stimulation. Data from a single representative experiment (**c**) and mean from 6 experiments (**d**).

Insets in **a** and **c** show the average of 60 evoked EPSPs before (gray) and 30 min after paradigm application (black). Gray vertical bars indicate the time intervals in which the induction paradigms were applied.

Supplementary Figure 4. STDP in CA3–CA3 synapses at near-physiological temperature.

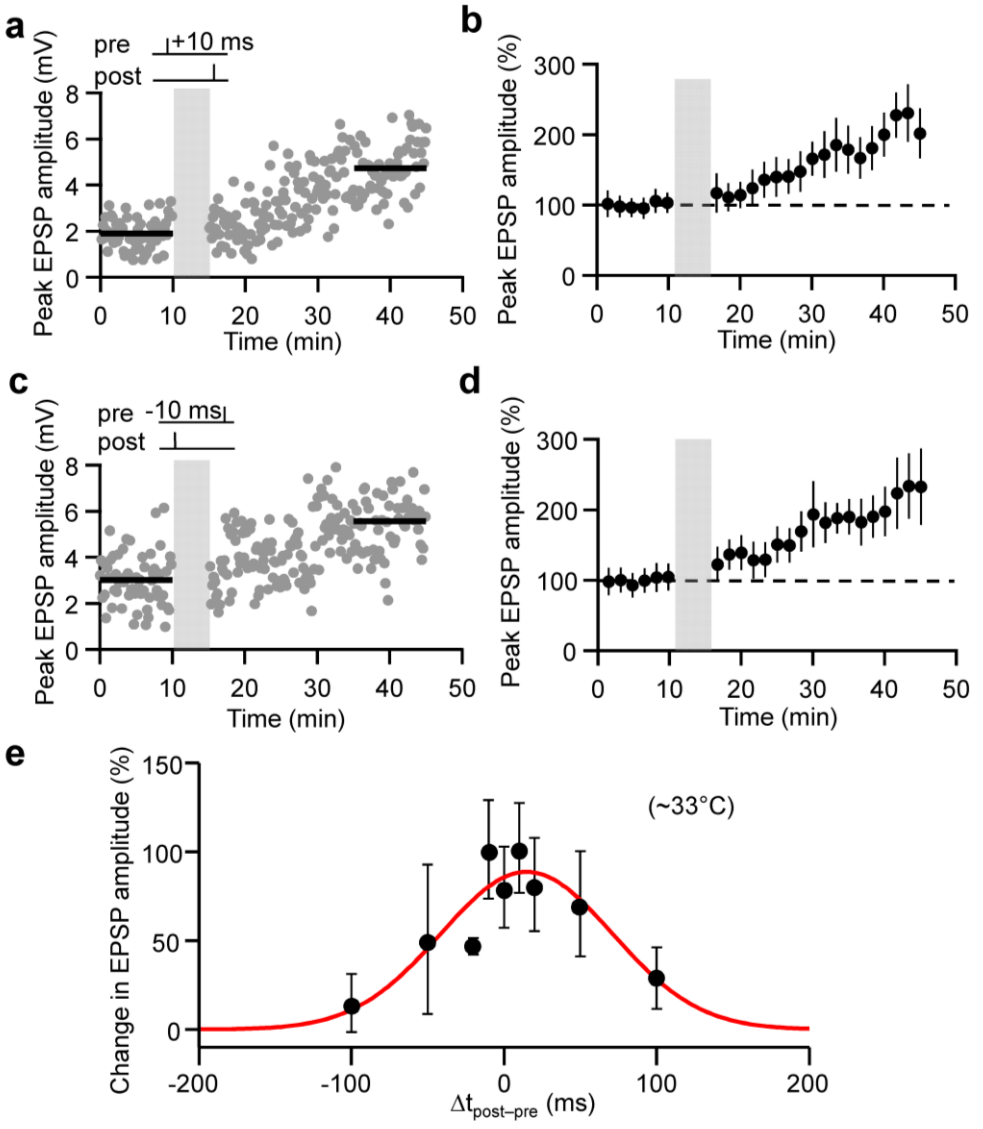

(a, b) Pre–postsynaptic pairing induces LTP at recurrent CA3–CA3 synapses at near-physiological temperature. Plot of compound EPSP peak amplitude against experimental time before and after pre–postsynaptic pairing (Δt = +10 ms). Single-cell data (**a**) and mean data (**b**; 6 cells).

(c, d) Post–presynaptic pairing also induces LTP at near-physiological temperature. Similar plot as in (**a, b**), but for post–presynaptic pairing (Δt = –10 ms). Single-cell data (**c**) and mean data (**d**; 5 cells).

(e) A broad and symmetric STDP curve at CA3–CA3 synapses at near-physiological temperature. Magnitude of potentiation induced by pre–postsynaptic and post–presynaptic pairing was plotted against pairing time interval Δt (34 cells total). Red curve, Gaussian function without offset fit to the data points. All data were obtained at ~33°C. Note that the STDP curve at ~33°C was broad and symmetric, similar to the STDP curve at room temperature (**Fig. 1i**).

Supplementary Figure 5. Properties of [Ca^{2+}] transients in CA3 pyramidal neuron dendrites and spines.

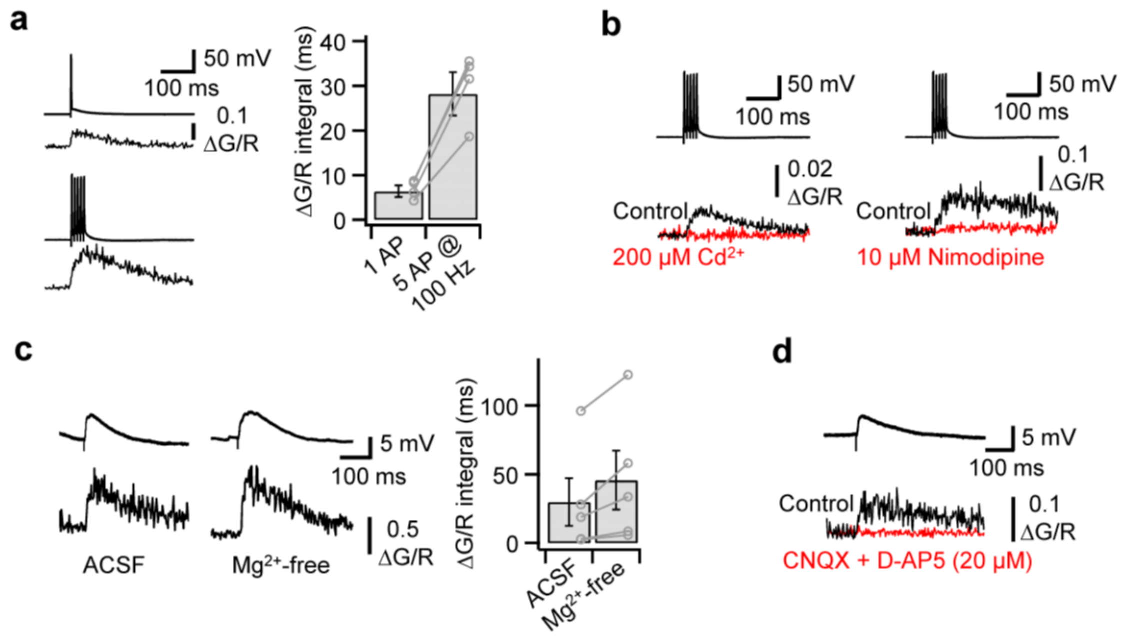

(a) Dendritic [Ca^{2+}] transients evoked by a single AP and a 100-Hz train of APs. Left, average [Ca^{2+}] transients. Right, summary bar graph of peak amplitudes of [Ca^{2+}] transients; data from the same experiment are connected by lines.

(b) Dendritic [Ca^{2+}] transients evoked by backpropagated APs were blocked by 200 µM of the broad-spectrum Ca^{2+} channel blocker Cd^{2+} (extracelluar, left) or 10 µM of the L-type Ca^{2+} channel blocker nimodipine (extracellular, right). Black traces, control; red traces, in the presence of blocker.

(c) Spine [Ca^{2+}] transients evoked by synaptic stimulation were increased in Mg^{2+}-free extracellular solution. Left, [Ca^{2+}] transients in standard ACSF; right, [Ca^{2+}] transients in Mg^{2+}-free solution. Right, summary bar graph integrals of [Ca^{2+}] transients in the two conditions; data from the same experiment are connected by lines.

(d) Spine [Ca^{2+}] transients evoked by synaptic stimulation were blocked by glutamate receptor antagonists (20 µM CNQX + 20 µM D-AP5). Black traces, control; red trace, in the presence of blockers.

Supplementary Table 1. Parameters of the pattern completion model.

Parameter	Explanation	Default value or range
n	Number of neurons	3,000
p	Connection probability	0.5
a	Total activity level	0.1
g_1	Inhibition factor	0 – 1
m	Pattern load (number of patterns applied in storage phase)	0 – 200
b_1	Proportion of valid firings in initial phase of recall (b_1 = 1 → identity to initial pattern)	0.5
b_n	Proportion of spurious firings in initial phase of recall (b_n = 1 → no spurious firing)	1
σ_t	Standard deviation of spike times in activity patterns	0.2 cycles
τ_{pot}	Synaptic potentiation time constant in the STDP rule	1 cycle
τ_m	Synaptic potential decay time constant	1 cycle

For details, see Methods and Gibson and Robinson, 1992; Bennett et al., 1994.

Supplementary References

1. Kamiya, H., Shinozaki, H. & Yamamoto, C. Activation of metabotropic glutamate receptor type 2/3 suppresses transmission at rat hippocampal mossy fibre synapses. *J. Physiol.* **493**, 447–455 (1996).
2. Gibson, W.G. & Robinson, J. Statistical analysis of the dynamics of sparse associative memory. *Neural Networks* **5**, 645–661 (1992).
3. Bennett, M.R., Gibson, W.G. & Robinson, J. Dynamics of the CA3 pyramidal neuron autoassociative memory network in the hippocampus. *Philos. Trans. R. Soc. Lond. B Biol. Sci.* **343**, 167–187 (1994).
